# Evaluations of modes of pooling specimens for COVID-19 screened by quantitative PCR and droplet digital PCR

**DOI:** 10.1038/s41598-024-61631-0

**Published:** 2024-05-13

**Authors:** Daitao Zhang, Lingyu Shen, Zhichao Liang, Shujuan Cui

**Affiliations:** Institute for Infectious Disease and Endemic Disease Control, Beijing Center for Disease Prevention and Control, Beijing Research Center for Respiratory Infectious Diseases, Beijing, 100013 China

**Keywords:** SARS-CoV-2, Pooled specimens, Swab pooling, Sampling swab, Virus preservation solution, Sensitivity, Immunology, Microbiology

## Abstract

Though pooling samples for SARS-CoV-2 detection has effectively met the need for rapid diagnostic and screening tests, many factors can influence the sensitivity of a pooled test. In this study, we conducted a simulation experiment to evaluate modes of pooling specimens and aimed at formulating an optimal pooling strategy. We focussed on the type of swab, their solvent adsorption ability, pool size, pooling volume, and different factors affecting the quality of preserving RNA by different virus solutions. Both quantitative PCR and digital PCR were used to evaluate the sampling performance. In addition, we determined the detection limit by sampling which is simulated from the virus of different titers and evaluated the effect of sample-storage conditions by determining the viral load after storage. We found that flocked swabs were better than fibre swabs. The RNA-preserving ability of the non-inactivating virus solution was slightly better than that of the inactivating virus solution. The optimal pooling strategy was a pool size of 10 samples in a total volume of 9 mL. Storing the collected samples at 4 °C or 25 °C for up to 48 h had little effect on the detection sensitivity. Further, we observed that our optimal pooling strategy performed equally well as the single-tube test did. In clinical applications, we recommend adopting this pooling strategy for low-risk populations to improve screening efficiency and shape future strategies for detecting and managing other respiratory pathogens, thus contributing to preparedness for future public health challenges.

## Introduction

Since January 2020, severe acute respiratory syndrome coronavirus 2 (SARS-CoV-2) has spread worldwide, seriously endangering public health and economic development. Due to the highly infectious nature of SARS-CoV-2, timely diagnosis and isolation of patients are the most effective ways to prevent the spread of the outbreak^1–5^. Currently, the primary method used to diagnose coronavirus disease 2019 (COVID-19) is the reverse transcription quantitative PCR (RT-qPCR), which is highly sensitive and specific but costly and relatively time-consuming^6–10^. Owing to limited resources, RT-qPCR testing is often limited to suspected patients with apparent symptoms. However, studies have shown that asymptomatic or mildly symptomatic patients can also transmit the virus^11^. Large-scale screening in communities, where patients have been diagnosed, can effectively control the spread of the virus^12,13^. However, large-scale screening poses severe challenges to the testing capacity of laboratories, and it is unaffordable for low-income countries with insufficient resources. A pooled test is an effective strategy to cope with this demand.

In practice, the pooled test of SARS-CoV-2^14^ mainly includes two methods, media pooling^11,15–20^ and swab pooling^21,22^. Media pooling is combining equal amounts of solution from multiple specimen tubes, while swab pooling is putting multiple swabs into a single tube, and this theoretically does not dilute the concentration of the virus. Lohse et al.^11^ and Hogan et al.^18^ conducted multiple media pooling tests and observed them as effective means for large-scale screening in addition to saving time and consumables. However, some studies suggest that due to the presence of negative specimens, media pooling reduces the detection sensitivity. Abdalhamid et al.^16^ tested 25 experimental media pools, each containing one SARS-CoV-2 positive specimen mixed with four negative specimens (50 μL each) for a total volume of 250 μL. They found that the cycle threshold (Ct) v Clinical evaluation of bacterial lues were higher than the original individual specimens within a range of 0–5.03 Ct. Swab pooling, which increases the number of swabs without increasing the volume of the preservation solution, did not dilute the virus concentration of positive samples. Christoff et al.^22^ compared pooled and individual samples from 613 patients and found no false negatives or false positives in the pooled tests. Chen et al.^21^ investigated the difference between the detection results of media/swab pooling and the individual test and concluded that media pooling significantly reduces the Ct value and that swab pooling does not affect the detection sensitivity. The U.S. Centers for Disease Control and Prevention pooling amendment gives details of media and swab pooling^23^ procedure used in different studies.

There are many confounding factors for swab pooling, such as normalization of the sampling process, viral load of the sample, RNA extraction efficiency^24^, and the sensitivity of the assay type. In this study, we examined the influence of swab numbers, type of sampling swab^25^, type of virus preservation solution, volume of the sampling solution, virus load of the positive cases and storage conditions of specimens on the sensitivity of a pooled test. We evaluated the sensitivity and feasibility of pooling specimens by RT-qPCR^26,27^. and droplet digital PCR (RT-ddPCR)^28^ to optimize the swab pooling strategy.

## Materials and methods

### Ethics statement

This study was reviewed and approved by the Ethics Committee of the Beijing Center for Disease Control and Prevention (2021026). Since these samples were initially sent for SARS-CoV-2 case confirmation and epidemic control, this study only collected control data without written informed consent. In addition, all methods were performed in accordance with the relevant guidelines and regulations by including a statement. Informed consent was obtained from all the participants.

### Sample collection and RNA extraction

Throat swabs used in the study was collected from healthy individuals with no respiratory symptoms and others who were unwell. To minimize the differences introduced by the sampling process, samples used in the study was collected by one individual, from one site, following the same collection procedure. The swabs were soaked in a virus preservation solution and immediately vortexed for 15 s. Viral RNA was extracted using KingFisher Flex (Thermo Scientific, Waltham, MA). The amount of each sample used was 200 µL and that of the elution solution was 60 µL. Two titers of Viral RNA standard materials were inactivated at 56 °C for 30 min, and confirmed by RT-qPCR according to the Prevention and Control Scheme of China, giving Ct values of N gene of 24.2 and 29.1.

### RT-qPCR and droplet digital PCR protocol

RT-qPCR was performed on the ABI Prism 7500 system (Thermo Scientific, Waltham, MA) by a 20 μL mixture that contained SARS-CoV-2 diagnostic assays (Kinghawk Pharmaceutical, Beijing, China) following the instructions detailed in the kit. Specifically, a 25 μL mixture is prepared, comprising the diagnostic assays along with 5 μL of nucleic acid (procured from the China National Institute of Metrology, model GBW(E)091089). The RT-qPCR kit detected opening reading frame (ORF) 1ab, the nucleocapsid protein (N) gene and RNase P (RNP), an internal reference gene in human epithelial cells, simultaneously. The RT-qPCR program was 50 °C for 30 min, then 95 °C for 3 min, and followed by 40 cycles at 95 °C for 10 s and 55 °C for 30 s. This adjustment is aligned with the kit's instructions and aims to enhance the reproducibility of the RT-ddPCR experiments.

The digital PCR (RT-ddPCR) system was the TD-1 Droplet Digital PCR System (TargetingOne, Beijing, China). RT-ddPCR was carried out with the SARS-CoV-2 nucleic acid detection kit (TargetingOne, Beijing, China) according to the manufacturer’s instructions. The master mix included 1× ddPCR supermix for probes, forward and reverse primers at 400 nmol/L, and GRAM+/GRAM− probes at 200 nmol/L. Each well was filled with a 1-µL sample of DNA and DNase/RNase Free water, resulting in a final volume of 30 µL. Following droplet generation, the samples were amplified with the following thermal cycling program: 55 °C for 15 min, 95 °C for 10 min, 45 cycles of 94 °C for 30 s and 57 °C for 1 min, and finally held at 12 °C for 5 min. The PCR amplification was performed on the A 300 instrument (LongGene, Hangzhou, China) with a 1.5 °C/s ramp. Finally, the thermally cycled samples were loaded onto the chip reader, and the data from cluster plots were analyzed by the analysis software.

### Study design

**Figure 1:**
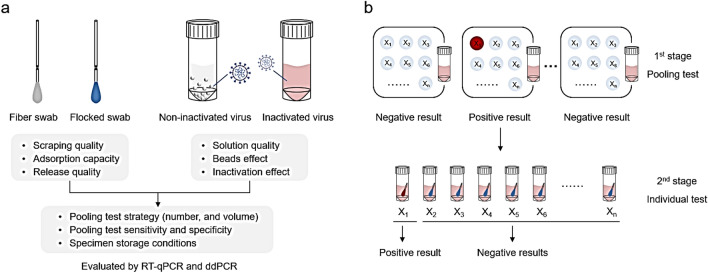
(**a**) Schematic diagram of the parameters tested. There were two types of swabs, fiber and flocked. And the properties tested for swabs were scraping quality, absorption capacity, and release quality. There were two types of preservation solutions, inactivating and non-inactivating. The solutions were tested for the solution quality, glass beads effect and whether the solutions are inactivated. Lastly, the pooling strategy (the number of swabs and the volume of preservations solution), the sensitivity and specificity of the pooled test, the performance of the preservation solution, and the influences of storage conditions were characterized by RT-qPCR and RT-ddPCR. (**b**) Schematic diagram of specimen pooling strategies. In the first stage of swab pooling, multiple swabs are pooled in a single sampling tube. If a positive sample is found, in the second stage of swab pooling, the attributors of swabs in the positive sample tube, need to be re-sampled and re-tested separately in order to find the corresponding positive sample.

As illustrated in the flowchart in Fig. 1a, we compared the performance of three brands of fibre swabs (labeled as Fib-1, Fib-2, Fib-3) and three brands of flocked swabs (labeled as Flk-1, Flk-2, Flk-3) in scraping quality, adsorption capacity, and release quality, respectively. We also tested six virus preservation solutions, including both three non-inactivating virus preservation solutions (labeled as NS-1, NS-2, NS-3) and three inactivating virus preservation solutions (labeled as IS-1, IS-2, IS-3) of different brands, to evaluate the effect of the pooled tests. Each experiment group had three replicates.

As shown in Fig. 1b, in the first stage, different numbers (the “n” in “X_n_”) of swabs were pooled in tubes to determine the optimal pool size and the volume of virus preservation solution to use. The sensitivity and specificity of the pooled test, and the preservation performance of the preservation solutions, were characterized by RT-qPCR and RT-ddPCR.

### Data analyses

Statistical analysis was determined by GraphPad Prism (GraphPad Software, San Diego, CA). Significant differences between the two groups were determined by the unpaired Student’s *t*-test. All reported p-values less than 0.05 was considered statistically significant, where “*” represents p < 0.05, “***” represents p < 0.001 and “ns” represents no significant difference.

## Results

### Comparison of sampling quality of different types of swabs

**Figure 2:**
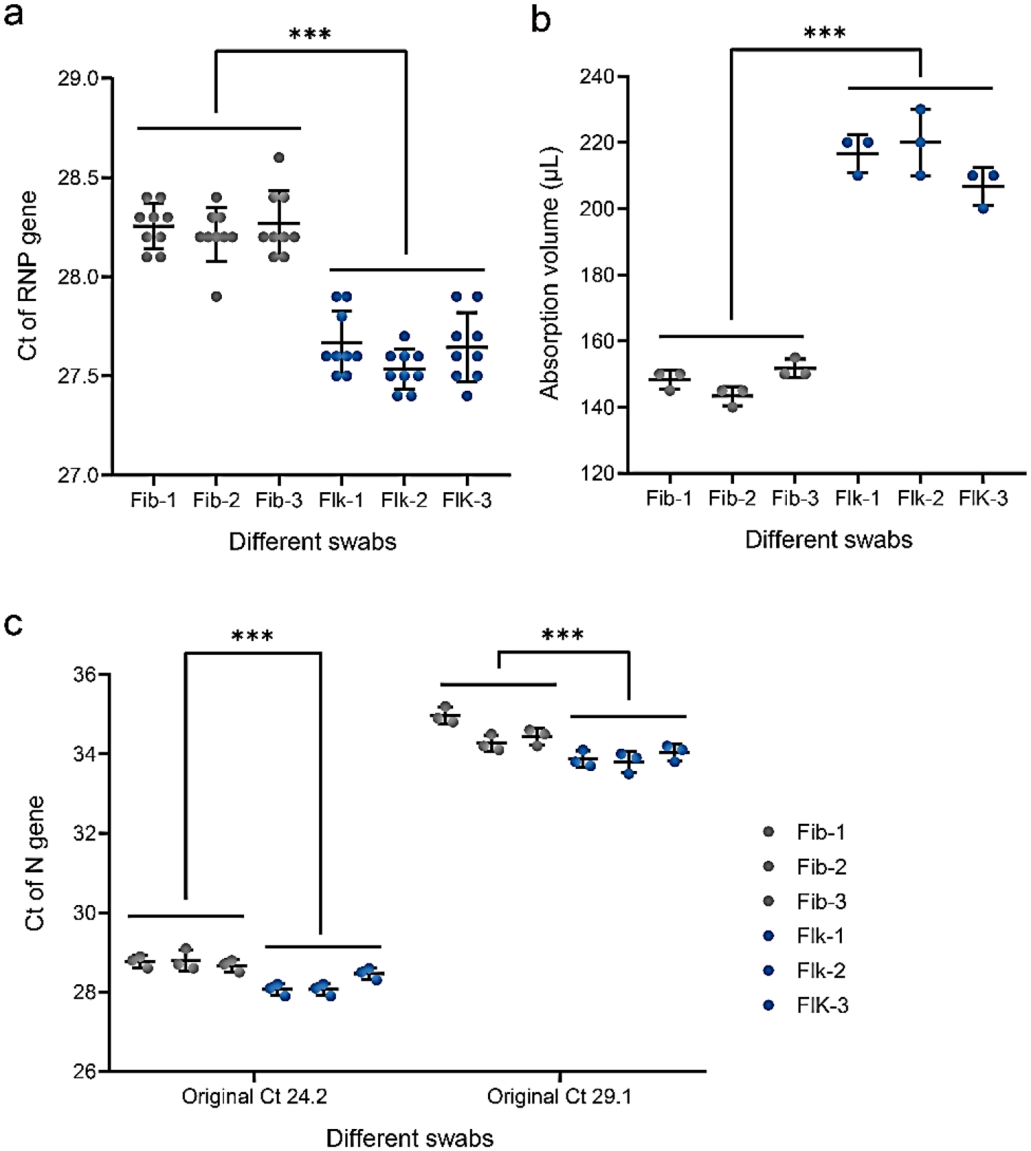
(**a**) Detection results of different fibre and flocked swabs in terms of the scraping quality. (**b**) Absorption volume of different fibre and flocked swabs. (**c**) Detection results of different fiber and flocked swabs that were soaked in two titers of viral RNA standard materials.

To compare the scraping quality of fibre swabs and flocked swabs, we collected throat swabs from healthy people with six brands of swabs, according to the pharyngeal swab sampling specification. The swabs were each placed in a sampling tube with 3 mL PBS. The sampling tube vortexed for 20 s, and then 200 µL was taken for nucleic acid extraction. The extracted nucleic acid was tested in triplicate by RT-qPCR. RNP quantification by RT-qPCR showed that the scraping quality of flocked swabs was better than fibre swabs (Fig. 2a).

To compare the adsorption capacity of different swabs, each swab was submerged in 3 mL PBS in a sampling tube for 10 s and then discarded. After centrifuging the sampling tube, we calculated the absorption volume by measuring the remaining volume of PBS. The average adsorption volumes of fibre swabs and flocked swabs were 148 μL and 214 μL, respectively, indicating that flocked swabs have better adsorption capacity (Fig. 2b).

Studies suggest that release quality of the swabs is closely related to the sensitivity of the test. To compare the release quality of different swabs, we immersed each swab in 100 µL viral standard materials with Ct = 24.2 and Ct = 29.1 and then placed it in 3 mL PBS in a sampling tube. We then vortexed the tube for 20 s, and took 200 µL for RNA extraction. The extracted RNA was quantified in triplicate by RT-qPCR for SARS-CoV-2. As shown in Fig. 2c, the release quality of the flocked swabs was superior to that of the fibre swabs, especially when the virus load was low.

### Comparison of the performance of different types of RNA virus preservation solutions

We selected two variables to determine the effect of the type of RNA virus preservation solution on the preservation of samples. First, whether the kit was inactivated; and second, whether there were glass beads in the virus preservation solution. Twelve identical flocked swabs were divided into two groups and immersed in two concentrations of viral standard materials. The swabs were then removed and placed in six virus preservation solutions: inactivating (IS-1, IS-2, IS-3) and non-inactivating (NS-1, NS-2, NS-3).

**Figure 3:**
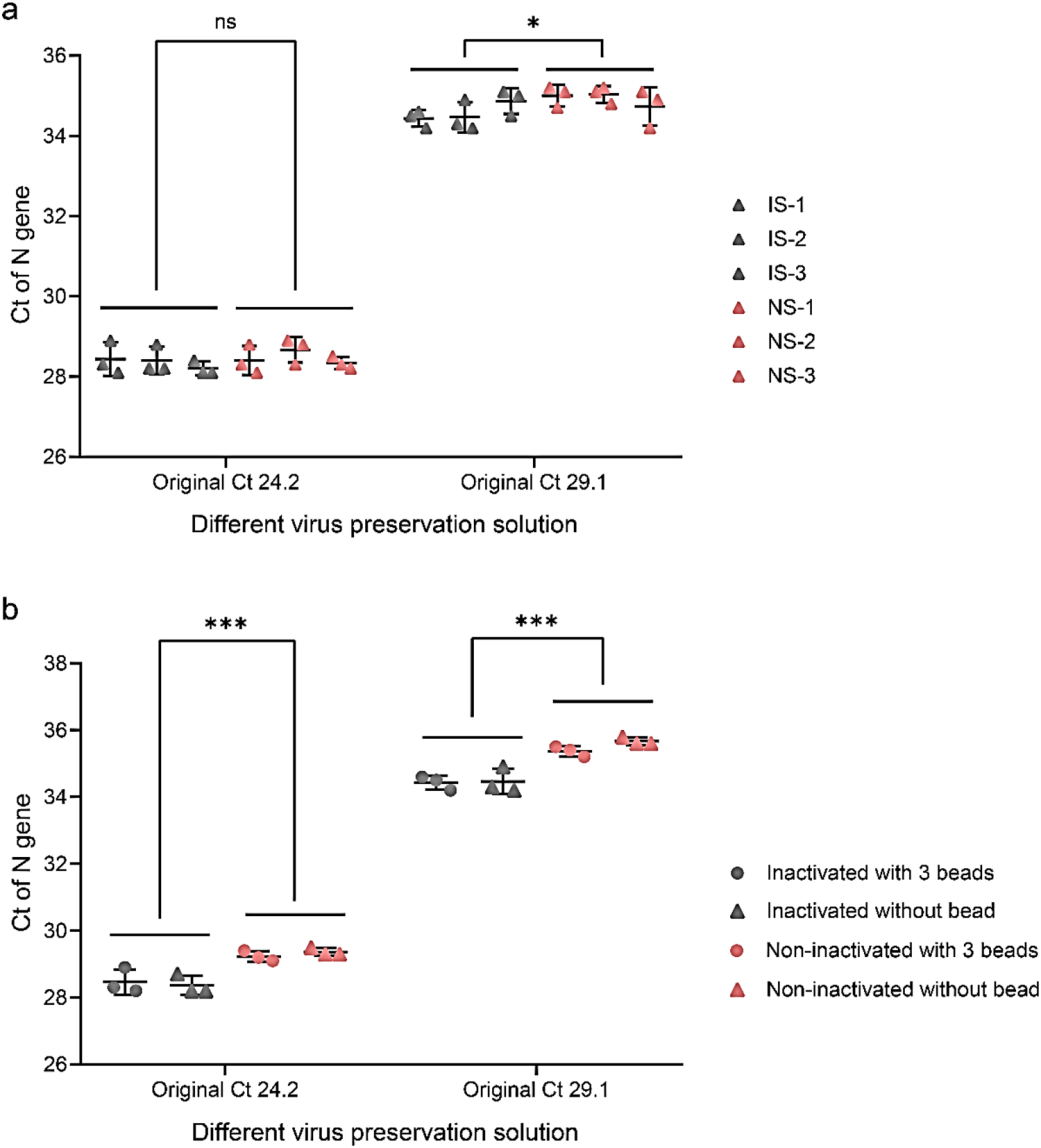
(**a**) Comparison of the preservation effect of inactivating and non-inactivating virus preservation solutions. (**b**) Effect of the presence of on the test results.

The Ct value was used to characterize the RNA preservation performance of the preservation solution. As shown in Fig. 3a, there was no significant difference between the non-inactivating and inactivating virus preservation solutions when the amount of virus on the sampled swab was high. When the amount of virus on the sampled swab was low, the non-inactivating solution had a slight advantage over the inactivating solution. The glass beads in the solution had no significant effect on the virus release from the swab, as shown in Fig. 3b.

### Determining the optimal number of swabs for swab pooling

Two brands of commercial SARS-CoV-2 RNA detection kits and two types of virus preservation solution were used to form four sets (labeled as XY-NS, XY-IS, YK-NS, YK-IS). Each set contained nine 50 mL centrifuge tubes with 15 mL of the virus preservation solution, one 10 mL centrifuge tube with 3 mL of the virus preservation solution, and a number of swabs, to determine the optimal number of pooled swabs for pooled test. Every set was used as follows: 10 swabs were completely immersed in the viral standard materials for 10 s and then placed in the 10 centrifuge tubes with virus preservative solution. Healthy individuals were sampled with swabs from the four sampling sets, and those collected swabs were used as negative samples. Different numbers of negative swabs were placed in each of the 10 centrifuge tubes containing one positive swab. The nine 50 mL centrifuge tubes were given 0, 2, 4, 6, 9, 11, 14, 16, and 19 negative swabs; and the one 10 mL centrifuge tube was not given any negative sample. The tubes were vortexed for 20 s, and 200 μL was removed for RNA extraction. The amount of virus was quantified by RT-qPCR.

**Figure 4:**
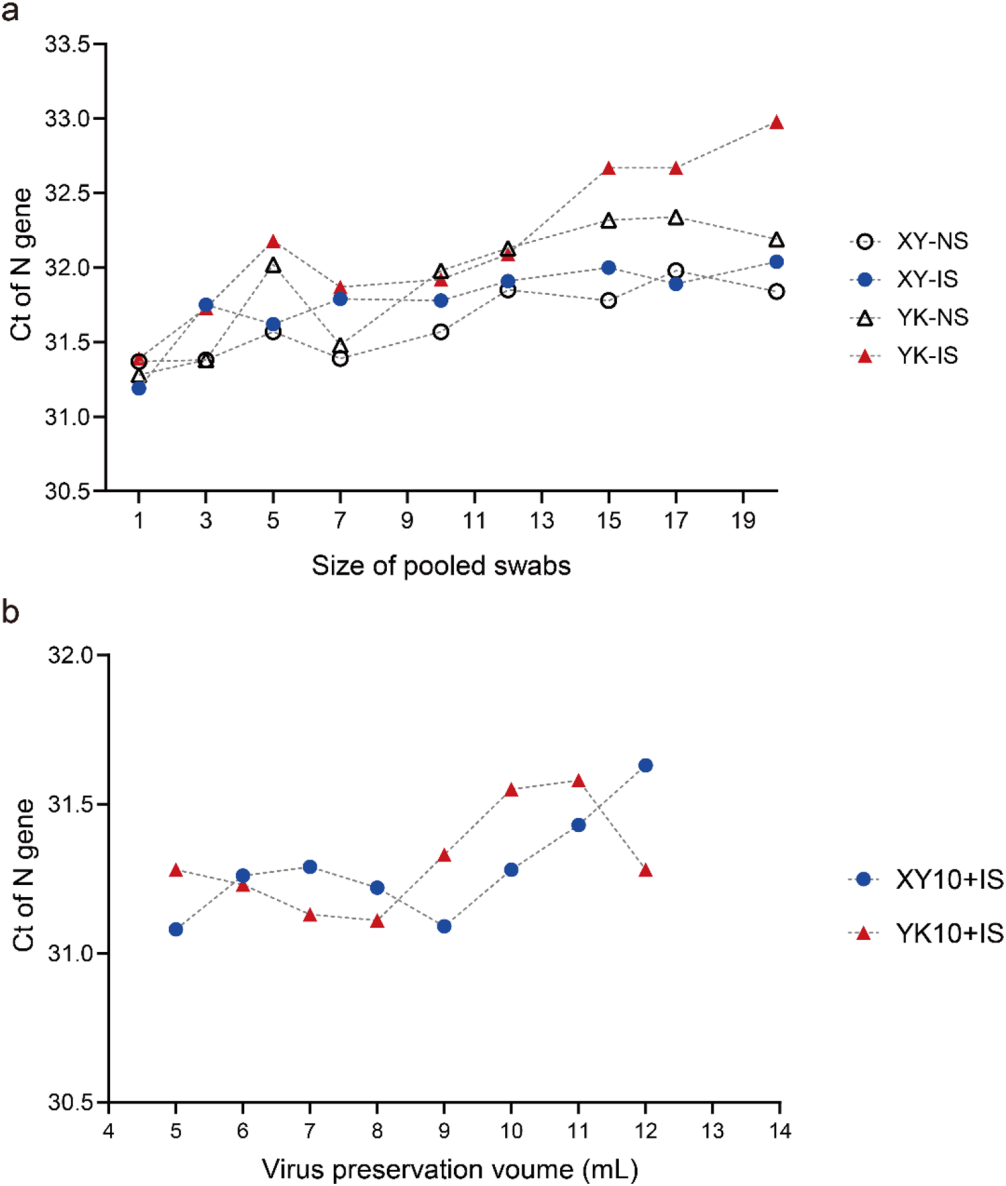
(**a**) Detection results of two brands of inactivating and non-inactivating virus preservation solutions with different numbers of pooled swabs. (**b**) Detection results of different volumes of virus preservation solution for 10-in-1 pooled swabs.

We focussed on two main criteria for selecting the optimal number of pooled swabs: first, a higher viral load detected (i.e., a smaller Ct value), indicating that the impact of pooling on the test sensitivity is small; second, a larger pool size when the detected Ct values are similar, which can save consumables and man-hours. As is demonstrated in Fig. 4a that when the number of swabs mixed was 10-in-1, the Ct value did not increase compared with a smaller pool size. The result of a pool size of 12 was larger than Ct 32, which may reduce the sensitivity in actual tests. Considering the above two factors, 10 pooled swabs was selected as the optimal pool size.

### Determining the optimal volume of virus preservation solution for swab pooling

The optimal volume of the two brands of virus preservation solution was determined on the basis of 10-in-1 pooled swabs. In the volume range from 5 to 17 mL, 7 groups of virus preservation solution were configured in 2 mL intervals and 10 swabs were added, one of which was a simulated positive sample swab soaked in a positive virus solution and the remaining nine were sampled healthy individuals. The pooled swabs were vortexed for 20 s, and 200 μL was removed for RNA extraction before detection by RT-qPCR. As is shown in Fig. 4b, the XY10 + IS specimen with 9 mL virus preservation solution had the lowest Ct value. Meanwhile, the YK10 + IS specimen with 8 mL virus preservation solution had the second lowest Ct value. Thus, 9 mL XY virus preservation solution was determined as the optimal volume for the swab pooling strategy.

### Determination of detection limit for swab pooling

Based on the optimal number of mixed swabs and the volume of virus preservation solution, the detection limit was determined by the number of viruses on individual positive swabs. Here, we used the RT-ddPCR method for accurate viral quantification. Our logic behind the use of RT-ddPCR for viral load detection and sensitivity was that this method gives absolute count of DNA molecules, eliminating the need to use and maintain reference material. The viral standard materials were diluted from 1000 copies/mL to 500/250/100/50/25/10 copies/mL. Swabs were completely immersed in a series of materials for 10 s and then placed in centrifuge tubes (50 mL volume) with 9 mL of virus preservation solution. Sixty-three swabs were used to sample healthy individuals and then placed into the centrifuge tubes described above, with 10 swabs per tube and vortexed for 20 s. Two hundred microliters were removed for RNA extraction, and then the amount of virus was quantified by RT-ddPCR to determine the detection limit. A positive swab at each virus dilution was added to 9 mL of virus preservation solution as a control.

Diluted viral materials from 1000 to 10 copies/mL were used to assess the sensitivity of pooling specimens. The limit of testing for pooling specimens was determined based on the threshold of RT-ddPCR. Based on the viral load of the single positive swab in 9 mL of viral solution, 50 copies/mL was found to be the test limit.

### Determination of storage conditions for swab pooling

**Figure 5:**
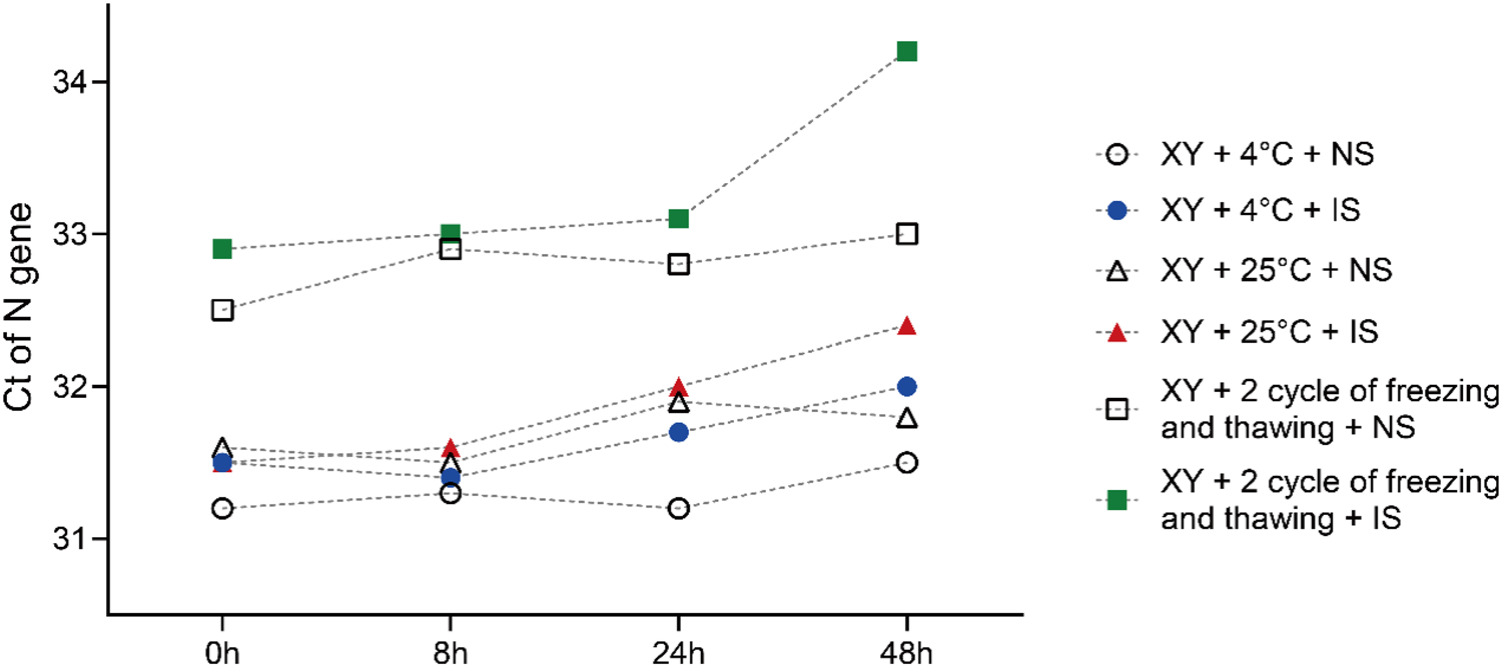
Assay results obtained for mixed samples under different storage conditions.

To analyze the effect of storage conditions on the pooled samples, we divided the samples in non-inactivating and inactivating virus preservation solution into three parts, two of which were placed at 4 °C and 25 °C, and the other one was placed at 4 °C after freeze-thawing twice. After 0 h, 12 h, 24 h and 48 h, 200 μL was removed from each sample for RNA extraction, and then the amount of virus was quantified by RT-qPCR. The results are shown in Fig. 5. The viral load detected in the samples decreased significantly after freeze-thawing twice. The samples stored at 4 °C produced better results than those stored at 25 °C.

### Sample validation for large-scale RT-qPCR screening

A number of 2342 low-risk individual samples were screened using the 10-in-1 pooled test, and no positive results had been tested. To evaluate the performance of the 10-in-1 pooled test, further, we collected 270 individual samples with epidemiological history of SARS-CoV-2, sampling 2 swabs at the same time, with one for a single-tube test and the other one for a 10-in-1 test. According to the RT-qPCR kit in a single-tube test, the Ct value less than or equal to 38 could be judged a positive result, which is regarded as a control. The 10-in-1 pooling test results show that all 262 negative samples and 7 positive samples could be correctly detected, though one of them was inconsistent with single-tube test results. Due to low viral load, the false-negative sample in 10-in-1 test Ct value greater than 38 and less than 40 was detected as the suspected sample, and it requires to be retested or sampled again.

## Discussion

The emergence of new SARS-CoV-2 variants with the waning of the pandemic has necessitated the need for accurate and rapid diagnostic and screening testing. In the context of a demand for SARS-CoV-2 testing in low-risk populations, swab pooling can improve screening efficiency and substantially reduce the need for sampling and testing kits while not considerably reducing the sensitivity of the test. However, pooling samples can be challenging, and various factors can influence the sensitivity of the screening procedure. Studies have shown that sensitivity decreases with increased pool size^29^. In addition, the efficiency of the pooled tests has been reported to depend on the viral load of the samples, the dilution of the sample^30^ and also the transportation mode, storage and sensitivity of the kit^31^. In this study, we analyzed the main factors that influence the performance of pooled tests. Our pool size was smaller than 20 since a larger pool size has been reported to impact the accuracy of the test results and place an excessive burden on the sampling procedure^30^. Pooling swabs for testing does come with potential drawbacks. We have listed them in the manuscript. In addition, we have also included our sample collection procedure used in this study to minimize the variations.

Sampling swabs and virus preservation solutions play an important role in collecting specimens^32^. Our study revealed that flocked swabs are superior to fibre swabs because of their consistent scraping ability, absorption capacity, and release ability. The differences between the swabs may be related to the different materials and manufacturing principles. Fibre swabs are bound in layers and are not conducive to scraping epithelial cells and releasing viruses^30^. Flocked swabs are made by an electrostatic flocking process that attaches millions of nylon microfibers vertically to the swab head, facilitating sample collection, keeping the sample on the swab surface, and allowing for easier virus elution^29^.

Using of a virus preservation solution ensures the integrity of the virus and facilitates the accuracy of subsequent tests^33^. We investigated two types of virus preservation solutions currently available on the market, inactivating virus preservation solutions and non-inactivating virus preservation solutions. Inactivating virus preservation solution inactivates and lyses the virus with guanidine salts. It protects the operator from a secondary infection by the virus while preserving the integrity of the nucleic acid^32^. The purpose of the non-inactivating virus preservation solution is to preserve the intact structure of the virus, not only for nucleic acid extraction but also for virus culture ^33^. Studies suggest that virus preservation solutions are almost always effective for virus detection, when the viral load is high. However, when the viral load is low, the non-inactivating virus preservation solution has a slight advantage. However, inactivating solutions can degrade viral RNA to some extent^27^.

In general, the bigger the pool size, the higher is the screening efficiency^27^. Considering the volume of the preservation tubes and swabs, we set the maximum number of mixed swabs at 20. To explore the optimal pool size and preservation solution volume, we collected products from major sample testing kit suppliers on the market and evaluated the pooled test performance using their swabs and virus preservation solutions. The number of swabs in the mix ranged from 1 to 20. To ensure that 20 swabs were fully immersed in the solution, we initially set the liquid volume of each tube to 15 mL, added only one positive swab per tube, and sampled the other swabs from healthy individuals. The healthy individuals in this study were those who had no clinical respiratory symptoms and was tested negative for other common respiratory pathogens detected by commercial kits.

Given the strong relationship between viral load and transmissibility of the SARS-CoV-2, accurate diagnostic testing is essential. Although quantitative reverse transcriptase PCR (qRT-PCR) has been recommended as the gold standard for COVID-19 diagnosis, it has been observed to have certain limitations. Studies have reported that the RT-qPCR results are limited by its reliance on a standard curve, sensitivity to inhibitors in clinical samples, and, more importantly, inconsistent performance at low concentrations. We used digital droplet PCR (RT-ddPCR) to determine the viral load from different pooled tests to assess the detection limit. Vasudevan et al. have demonstrated that RT-ddPCR can robustly quantify SARS-CoV-2 RNA from crude viral lysate^31^. From the viral load RT-qPCR results of the different pooled tests, we learned that the sensitivity of the assay has been guaranteed, and the efficiency of the assay has been improved when the number of pooled swabs is 10.

In determining the optimal volume of the virus preservation solution, we set up the volume in the 5 to 17 mL range at 2 mL intervals. Our results showed that 9 mL of virus preservation solution was optimal. The 5 mL pool volume could not thoroughly soak 10 swabs. Consequently, it could not completely release the virus on the swabs into the solution. For volumes greater than 9 mL, although the virus on the swab can be better released into the solution, it may dilute the virus concentration due to the larger volume, thus reducing the sensitivity of virus detection.

Although a larger pool size can improve the screening efficiency, it can also reduce the sensitivity of the assay^30^. Thus, we preferred 10 swabs in one preservation tube and 9 mL of preservation solution. Next, we proceeded to determine the limit of detection for this strategy. The final limit of detection was determined to be 50 copies/mL. It is crucial to perform a diagnostic analysis of the Ct value or copies of the test results during the implementation of pooled tests. In this study, we used both RT-qPCR and RT-ddPCR for evaluating of swab performance, viral load, and RNA preservation.

While screening large numbers of samples, it takes a long time to collect samples from the local field and transport them to the laboratory. This process prolongs the duration of the overall nucleic acid test. Under certain specific conditions, improper storage conditions of the samples may affect viral load. Therefore, we evaluated the effect of placing samples at different temperatures. The results showed little difference in viral load over 24 h storage at 4 °C and 25 °C. After 48 h, the result of 4 °C storage was slightly better than that of 25 °C storage. Therefore, sample storage for 48 h should have little effect on the sensitivity of the assay.

To reduce the influence of different sampling personnel on sample collection, these samples were taken by the same doctor, from the same collection site, with the same collection steps, and the same vortexing time, to minimize the differences introduced by the sampling process. If more sampling personnel are required, they would be rigorously trained with the same sampling procedures and quality controls^30,34^. The main drawback of our study is that it was conducted in a defined geographical area, and a viral load was adjusted experimentally or in standard mode used for adjusting^35^. in the laboratory. More studies and data from different geographical regions and samples from other SARS-CoV-2 variants risk areas are needed to validate our sample pooling strategies. In addition, we can expand the scope of such a study to assess its applicability to other respiratory viruses such as RSV and influenza. However, it's important to note that the effectiveness of pooling strategies can vary depending on the specific characteristics of each virus, including its stability, shedding patterns, and prevalence in the population. Therefore, while this research proposed pooling strategy may serve as a useful starting point, it would be prudent to conduct similar evaluations and optimizations tailored to the unique properties of RSV and influenza before widespread adoption. Nonetheless, this research stands as a promising foundation for future exploration and adaptation in the context of these viruses.

In conclusion, our study reveals that flocked swabs are a better choice than fibre swabs, 10 swabs pooled in one tube of 9 mL preservation solution gives better results, samples stored at 4 °C or 25 °C for up to 48 h has no significant effect on the sensitivity of virus detection, and virus preservation solutions do not have any effect on the efficiency and sensitivity of RT-qPCR or RT-ddPCR. We, therefore, recommend adopting a 10-in-1 pooling test for low-risk populations. If a positive result is found, the single-tube detection method can be used to track the epidemiological source.

## Data Availability

The datasets used and/or analyzed during the current study are available from the corresponding author on reasonable request.
